# Adaptive Evolution of *Pseudomonas aeruginosa* in Human Airways Shows Phenotypic Convergence Despite Diverse Patterns of Genomic Changes

**DOI:** 10.1093/molbev/msae022

**Published:** 2024-02-14

**Authors:** Akbar Espaillat, Claudia Antonella Colque, Daniela Rago, Ruggero La Rosa, Søren Molin, Helle Krogh Johansen

**Affiliations:** Department of Clinical Microbiology 9301, Rigshospitalet, Copenhagen Ø 2100, Denmark; Department of Clinical Microbiology 9301, Rigshospitalet, Copenhagen Ø 2100, Denmark; The Novo Nordisk Foundation Center for Biosustainability, Technical University of Denmark, Lyngby 2800, Denmark; Department of Clinical Microbiology 9301, Rigshospitalet, Copenhagen Ø 2100, Denmark; The Novo Nordisk Foundation Center for Biosustainability, Technical University of Denmark, Lyngby 2800, Denmark; The Novo Nordisk Foundation Center for Biosustainability, Technical University of Denmark, Lyngby 2800, Denmark; Department of Clinical Microbiology 9301, Rigshospitalet, Copenhagen Ø 2100, Denmark; Department of Clinical Medicine, Faculty of Health and Medical Sciences, University of Copenhagen, Copenhagen N 2200, Denmark

**Keywords:** evolution, adaptive evolution, cystic fibrosis, *Pseudomonas aeruginosa*, clinical isolates

## Abstract

Selective forces in the environment drive bacterial adaptation to novel niches, choosing the fitter variants in the population. However, in dynamic and changing environments, the evolutionary processes controlling bacterial adaptation are difficult to monitor. Here, we follow 9 people with cystic fibrosis chronically infected with *Pseudomonas aeruginosa*, as a proxy for bacterial adaptation. We identify and describe the bacterial changes and evolution occurring between 15 and 35 yr of within-host evolution. We combine whole-genome sequencing, RNA sequencing, and metabolomics and compare the evolutionary trajectories directed by the adaptation of 4 different *P. aeruginosa* lineages to the lung. Our data suggest divergent evolution at the genomic level for most of the genes, with signs of convergent evolution with respect to the acquisition of mutations in regulatory genes, which drive the transcriptional and metabolomic program at late time of evolution. Metabolomics further confirmed convergent adaptive phenotypic evolution as documented by the reduction of the quorum-sensing molecules acyl-homoserine lactone, phenazines, and rhamnolipids (except for quinolones). The modulation of the quorum-sensing repertoire suggests that similar selective forces characterize at late times of evolution independent of the patient. Collectively, our data suggest that similar environments and similar *P. aeruginosa* populations in the patients at prolonged time of infection are associated with an overall reduction of virulence-associated features and phenotypic convergence.

## Introduction

Microbial adaptation to a particular environment is directed by specific selective forces, and each niche represents a unique fitness landscape for the infecting bacteria. In this scenario, the selection of beneficial mutations that fix and expand in the population helps bacteria to successfully adapt and persist. Yet, monitoring the occurring adaptational events in dynamic natural environments and inferring the driving selective pressures remain a challenge, due to (i) complex spatial–temporal fluctuating conditions (e.g. temperature, pH, osmolality, and nutrient gradients), (ii) population dynamics (e.g. prey–predator, mutualistic relationship, or pathogenic interactions), and (iii) interkingdom interactions (host–microbe interactions). Genetic variations in such bacterial populations have, therefore, been difficult to associate with specific adaptive processes, if the complex conditions are at least not transiently stable. Consequently, mutation acquisition as a proxy for the selective pressures is usually insufficient to validate the particular significance of the specific genetic modifications (Rossi et al. 2021).

We investigate bacterial adaptation and evolution of *Pseudomonas aeruginosa* (*Pa*) in the airways of people with cystic fibrosis (pwCF) during the progression of colonization and infection. The CF lung infection model offers unique opportunities, as there is extensive within-patient follow-up information. CF sputum samples are routinely sampled from patient cohorts to diagnose bacterial infection status, and detailed characterization of CF lung disease progression has been well documented (Bhagirath et al. 2016; Khan et al. 2019). Considering the complexity and dynamics of the human airways, including the particular multispecies microbial communities described in CF airways, we suggest that our findings concerning bacterial adaptation in this environment reflect evolutionary processes occurring generally in many other natural environments (Yang et al. 2011; Damkiær et al. 2013).

Previously, the evolutionary dynamics of a persistent and highly successful *Pa* lineage, DK02, that had disseminated to more than 40 patients in the Copenhagen CF clinic, has been described (Yang et al. 2011), with respect to the genomic and phenotypic changes over a period of more than 200,000 bacterial generations (Yang et al. 2011). The DK02 lineage shows limited interpatient diversification, after an initial period of rapid adaptation. This is most likely caused by the acquisition of a few regulatory mutations affecting the transcriptional profile followed by a period of genetic drift with minor transcriptional changes (Yang et al. 2011; Damkiær et al. 2013).

This opened the question of whether the DK02 evolutionary history could be used as a reference to predict the adaptive pathways for other *Pa* lineages, when adapting toward a state of chronic infections in human airways. To address this question, we here investigate the evolution of 3 alternative and widespread persistent *Pa* lineages, DK01 (Markussen et al. 2014), DK19 (PA14) (Cramer et al. 2011; Mathee 2018), and DK06 (C-clone) (Römling et al. 2005; Lee et al. 2020), in comparison with the DK02 evolutionary pathway. Specifically, we resolved the evolutionary history of *Pa* in the airways of several chronically infected pwCF, as they reflect an adapted population to the lung environment. As we have previously shown that comprehensive collections of single isolates can infer the evolutionary dynamics of a diversified population (Sommer et al. 2016), we therefore focus our efforts on single isolates and their persistence over extended periods of times (≥15 yr) in different pwCF. The strategy has been to correlate the acquired mutations identified in the respective genomes with the globally expressed transcriptional network in DK02 and the resulting biosynthetic products. Overall, we document the value of using combinations of omic approaches to better understand evolutionary dynamics in complex environments.

## Results

### Within-Patient Genome Evolution: Divergence and Convergence of *Pa* Lineages

**Fig. 1.**
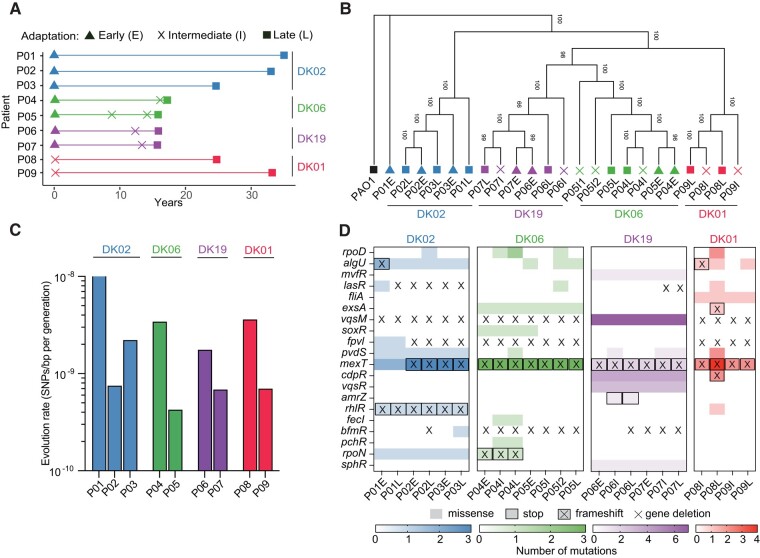
Distribution of the lineage-associated mutations. a) Longitudinal isolated samples from pwCF. Lineages dominating each patient are color coded (DK01 red, DK02 blue, DK06, green, and DK19 purple). b) Evolutionary history of the isolates represented by maximum likelihood reconstruction. The bootstrap (500 replicates) tree is based on the concatenated SNPs of each isolate relative to PAO1. c) Estimation of evolution rates as SNPs per base pair per generation time. d) Heatmap of the mutations found within regulatory genes.

To characterize the evolutionary trajectories securing the persistence of *Pa* in pwCF, we investigated a collection of Pa clinical isolates sampled longitudinally between 1973 and 2021 from 9 patients attending the Copenhagen CF Clinic (Table 1). We selected lineages based on (i) high prevalence in both Danish and international pwCF and (ii) with evolutionary histories spanning more than 15 yr. The lineages that were selected, DK01 (pwCF, *n* = 30), DK02 (pwCF, *n* = 35), DK06 (pwCF, *n* = 9), and DK19 (pwCF, *n* = 9), are in the top 10 most-abundant clone types from the Copenhagen CF Clinic (supplementary fig. S1, Supplementary Material online). Despite the presence of a heterogeneous population with high diversity in each pwCF, we analyzed single isolates representing the most abundant *Pa* representative of a sputum sample. Indeed, we previously spotlighted that single isolates can well represent the infecting population of a pwCF, therefore allowing us to characterize the adaptive and evolutionary process of *Pa* in pwCF (Sommer et al. 2016). For each patient, we compared initial isolates (referred to as “early”) collected within 2 yr of the diagnosis of chronic infection, with isolates collected after 15 yr (DK06 and DK19 lineages) and 35 yr (DK02 lineage) (referred to as “late”) of infection (Fig. 1a; Table 1). For the DK01 lineage, no early isolates were available in our collection, and therefore, “intermediate” strains isolated more than 10 yr after the onset of chronicity and evolved for 35 yr in each patient were used (Fig. 1a; Table 1). This collection comprises isolates with evolutionary histories estimated to cover between 35,000 and 150,000 bacterial generations (supplementary table S1, Supplementary Material online).

**Table 1:** Overview of *Pa* sampling from the different CF patients used in this study

Isolate	Lineage	State	Date of sampling	Years of evolution^a^
**P01E**	DK02	Early	1973	0
**P01L**	DK02	Late	03/01/2008	35.0
**P02E**	DK02	Early	1984	0
**P02L**	DK02	Late	07/01/2017	33.0
**P03E**	DK02	Early	1991	0
**P03L**	DK02	Late	26/08/2015	24.6
**P04E**	DK06	Early	04/02/2004	0
**P04I**	DK06	Intermediate	19/02/2020	16.0
**P04L**	DK06	Late	16/03/2021	17.1
**P05E**	DK06	Early	06/09/2006	0
**P05I1**	DK06	Intermediate	01/01/2014	8.8
**P05I2**	DK06	Intermediate	16/05/2019	14.0
**P05L**	DK06	Late	12/01/2021	15.7
**P06E**	DK19	Early	04/07/2006	0
**P06I**	DK19	Intermediate	12/09/2017	12.2
**P06L**	DK19	Late	16/03/2021	15.7
**P07E**	DK19	Early	05/04/2006	0
**P07I**	DK19	Intermediate	23/10/2018	13.1
**P07L**	DK19	Late	22/02/2021	15.4
**P08I**	DK01	Intermediate	1984	0
**P08L**	DK01	Late	2009	25.0
**P09I**	DK01	Intermediate	1984	0
**P09L**	DK01	Late	23/06/2016	32.80273973

^a^The years of evolution for an isolate within the patient were calculated as the isolation date between early and that of intermediate/late isolate.

Single nucleotide polymorphisms (SNPs) were used as phylogenetic markers to reconstruct the evolutionary history of the strains. As previously observed (Marvig et al. 2015), the genomes grouped primarily according to their lineage, suggesting a strong evolutionary contingency (Fig. 1b). Since niche specialization depends on the constant modulation of the mutation rate (Denamur and Matic 2006), we evaluated if the genome-wide mutation rates in the lineages differed. Despite significant differences in the numbers of generations, neither the synonymous nor the nonsynonymous mutation rates were found to differ between lineages (Fig. 1c; supplementary table S1, Supplementary Material online). Similarly, the mutation frequency to rifampicin showed no differences between lineages (ANOVA *P* > 0.05) even though strain DK01L-P08L showed increased mutation rate (27-fold) relative to PAO1 (supplementary table S2, Supplementary Material online). Of note, this isolate harbored 2 missense mutations in DNA mismatch repair gene *mutL*, one of the most common causes of a hypermutator phenotype in isolates from pwCF (Oliver et al. 2000). In this case, these mutations did not cause an overall increase in the genome mutation frequency, perhaps due to being just above the phenotype hypermutator limit (>20-fold). As expected, the minimum inhibitory concentrations (MICs) for antibiotics used as primary anti-*Pa* antibiotics (tobramycin, ciprofloxacin, and ceftazidime) increased between pair of early/intermediate and late isolates, albeit only a few isolates presented clear clinical antibiotic resistance (supplementary table S2, Supplementary Material online).

When comparing the introduced genetic changes across lineages, we found evidence for both convergent and divergent evolution within and between lineages. Among the mutated genes identified in the early/intermediate isolates, we found ∼15% of these to be shared across the samples (supplementary fig. S2a, Supplementary Material online). Specifically, all lineages showed convergent acquisition of mutations in genes related to (i) virulence pathways (e.g. Type II and III secretion apparatus and iron homeostasis), (ii) antibiotic resistance (e.g. efflux pumps systems), and (iii) motility (supplementary table S3, Supplementary Material online). These traits are known to be frequently lost after the establishment of chronic *Pa* infections in CF airways (Rossi et al. 2021). When comparing the late isolates, ∼32% of the mutated genes were shared (supplementary fig. S2a and table S3, Supplementary Material online). All late isolates shared mutations in (i) several TonB-dependent receptors related to the uptake of different molecules, (ii) multiple 2-component sensor regulators, and (iii) mutations in components or effectors of the Type VI secretion system (supplementary table S3, Supplementary Material online). Among the accumulated mutations from early/intermediate to late isolates, only low percentages (∼2% for DK01 and DK02, 6% for DK06, and 24% for DK19) of mutated genes were shared within each specific lineage. In contrast, similar categories of genes and biological pathways were shared across the different lineages (supplementary fig. S2b and c, Supplementary Material online). Overall, our data suggest that early mutational patterns show partial convergence at the genomic level, whereas at later stages of infection, there is a strong within-patient specialization of the particular isolate/lineage (genomic divergence).

The repeated occurrence of pathoadaptive mutations suggests convergent mechanisms of adaptation at both the genomic and the phenotypic levels (Yang et al. 2011; Marvig et al. 2015; Dettman and Kassen 2021). Interestingly, regulatory mutations show evolutionary convergence, either in a lineage-independent manner (fixed in all lineages) or with reference to time (early/intermediate → late), patient, or lineage (Fig. 1d; supplementary table S4, Supplementary Material online). In the case of the lineage-independent mutations, missense and/or frameshift mutations were identified in the genes encoding the multidrug efflux pump regulator MexT and in the virulence modulator VqsM (Fig. 1d; supplementary table S4, Supplementary Material online). Similarly, *fpvI* encoding the sigma factor and master regulator of iron homeostasis displayed genetic variations in all DK01, DK02, and DK06 isolates. Interestingly, in intermediate and late isolates of DK19, mutations were observed in the *pvdS* regulator gene, which belongs to the same regulatory network as *fpvI* (Fig. 1d; supplementary table S4, Supplementary Material online). The *bfmR* regulator (involved in biofilm maturation, Rhl quorum-sensing [QS] system, and active in acute infections) and the *algU* regulator (involved in alginate biosynthesis) show a certain degree of convergent evolution being mutated in more than half of the isolates. The antisigma factor, *mucA*, which modulates the activity of *algU* and causes a mucoid phenotype displayed by several *Pa* isolates, showed time-dependent frameshift mutations in all late isolates (supplementary table S5, Supplementary Material online). Several additional regulators such as *mvfR*, *lasR*, *fliA*, *exsA*, *cdpR*, *vqsR*, *rhlR*, *rpoN*, and *sphR* showed lineage-dependent convergence confirming strong evolutionary contingency between lineages (Fig. 1d; supplementary table S4, Supplementary Material online).

In summary, convergent evolution was observed for 3 categories of master regulators controlling envelope remodeling (*mucA*-*algU*), iron metabolism (*fpvI* and *pvdS*), and QS virulence modulation (*lasR*, *rhlR*, *vqsM*, *mexT*, and *bfmR*) (Fig. 1d). Our analysis suggests that common selective forces drive the acquisition of mutations in selected regulatory networks in a patient-independent manner. In addition, evolutionary contingency selects for lineage-dependent variants favoring adaptation to the patients.

### Transcriptional convergence of *Pa* lineages

We previously suggested that the acquisition of several regulatory mutations converted DK02 into a lineage highly adapted to the human airways (Damkiær et al. 2013). Limited transcriptional changes were, indeed, observed upon acquisition of mutations affecting the envelope (*algU*), catabolism (*rpoN*), and QS (*lasR-rhlR*), even after 3 decades of infection. To investigate the transcriptional impact of the lineage-specific and shared mutations identified in the DK02 lineage, we performed RNA-sequencing (RNA-seq) analysis under conditions mimicking the metabolic conditions in CF [e.g. Synthetic Cystic Fibrosis Medium (SCFM)].

**Fig. 2.**
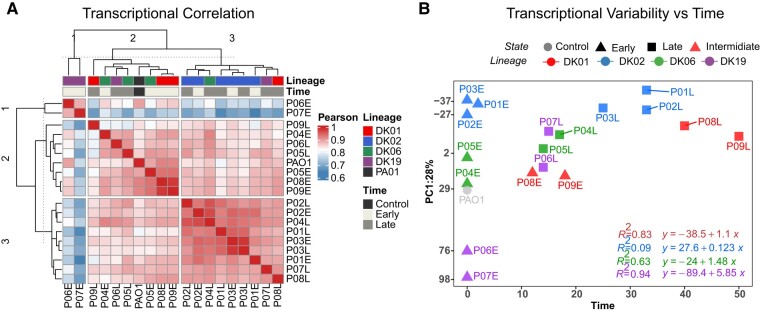
Transcriptional variability over time. a) Transcriptional-based correlation expressed as Pearson's correlation coefficient (*r*) and visualized as a heatmap of transcriptional profiles. b) Unsupervised PCA (PC1) loadings for each sample graphed as a function of time. PC1 represents 39% of the explained variability. Lineages dominating each patient are color coded (DK01 red, DK02 blue, DK06, green, and DK19 purple).

Pearson correlation analysis applied to the expression data (normalized reads) showed that except for DK02 isolates, the transcriptional correlation among the samples was dependent on the *time of evolution* (early/intermediate → late transition), rather than specific for each *lineage* (Fig. 2). As previously demonstrated (Bhagirath et al. 2016), all DK02 isolates showed a strong correlation coefficient (Pearson's correlation coefficient > 0.95), independent of time of evolution. Moreover, 1 late isolate from each lineage clustered with the DK02 transcriptomes suggesting convergent evolution at late times after the onset of chronic infection (Fig. 2a). In contrast, the early isolates of DK19 clustered as a very distinctive class separated from all the samples indicating a very distinctive transcriptional profile at early starting points (Fig. 2a). A similar result is obtained when comparing transcriptional profiles using principal component analysis (PCA). All late *Pa* strains cluster closer to the DK02 strains rather than their specific early strains indicating convergent evolution (supplementary fig. S3a, Supplementary Material online).

Since the time of evolution (years after the chronic infection was diagnosed) seems to play a major role in shaping the lineage phenotype, we compared the transcriptional variability to the length of infection by performing PCAs on the normalized reads versus time (Fig. 2b). The more evident signature was for DK19, for which the transcriptional variability over time (assessed by slope regression, *R*^2^ of 0.95) was 5- to 6-fold higher than that of the other lineages—followed by DK06 and DK01, with an *R*^2^ of 0.63 and 0.83, respectively (Fig. 2b). DK02 displayed an *R*^2^ and slope close to 0, representing essentially no transcriptional changes during the lineage evolution (Fig. 2b). In contrast, the rest of lineages seem to reflect phenotypic transition states directed toward the stable transcriptional state observed for DK02 (Fig. 2b).

To illustrate the impact of the specific regulatory mutations on the transcriptional network of the DK02 lineage, we included 2 PAO1 derivative mutant strains harboring the same mutations as the early DK02 isolates. Specifically, strain “RegMut” harbors alterations in *mucA-algT-rpoN*, while strain “RegMutΔlasR” harbors an additional deletion in *lasR* regulator (Damkiær et al. 2013). Both PCA and Pearson correlation analysis showed that such strains represent late transcriptional states close to those of the DK02 isolates (supplementary fig. S3a and b, Supplementary Material online), suggesting that the transcriptional state and stability seen already in early isolates of DK02 represent an adaptive maximum, which many/all *Pa* strains attain over time after infection of the human airways.

### Pathway Selectivity at Late Time Points

**Fig. 3.**
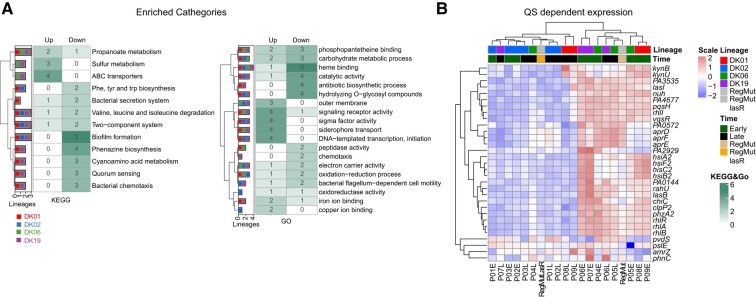
Convergent evolution on different lineages. a) KEGG and GO enrichment counts of the early–late stage are represented as a heatmap of the sum classes. b) Transcriptional patterns of DEGs of *lasR* regulon. Expressed as log-regularized read counts, scaled for each row, and visualized as a heatmap. Each column represents an analyzed sample and is clustered based on the result of pvclust. Lineages dominating each patient are color coded (DK01 red, DK02 blue, DK06, green, and DK19 purple).

To further characterize the transcriptional changes associated with the late chronic adaptation states, we identified differentially expressed genes (DEGs) in early/intermediate versus late strains and performed enriched analysis based on KEGG (Kyoto Encyclopedia of Genes and Genomes) / GO (Gene Ontology) pathway / function classifications. This provided identification of selective outcomes from the fixed mutations and characterization of their influence on the directionality of the transcriptional program. KEGG and GO enrichment analysis showed that during the adaptive processes, different lineages displayed similar enriched pathways and thus, evolutionary convergence (Fig. 3a; supplementary fig. S4, Supplementary Material online). The lineages converged at late time with higher frequency in 3 major features: (i) increase in expression of ABC transporters for sulfur metabolism, (ii) decrease in expression of QS regulators controlling, e.g. cyano-amino acid metabolism and phenazine biosynthesis, (iii) decrease in expression of certain biofilm biosynthetic genes, (iv) activation of transcriptional factors related to siderophore uptake/activation (Fig. 3a; supplementary fig. S4, Supplementary Material online).

Most of these time-associated changes are stably conserved in the DK02 lineage or present a time-dependent regulation (e.g. biofilm downregulation), once more suggesting that all isolates of the DK02 lineage are fully adapted at an early infection stage (supplementary fig. S4, Supplementary Material online). Importantly, our data further suggest that the enrichment of certain mutations at later infection stages may be related to pathway selectivity, showing cases of convergent genomic evolution among different lineages. Furthermore, depending on the evolutionary state of the specific isolate, our data highlight the strong selective pressure for the modulation of QS, as many of the convergent changes are regulated by it (e.g. biofilm, siderophore, and phenazine) (Fig. 3b).

Comparing the transcriptional levels of genes regulated by QS in early and late isolates suggests that QS exerts strong negative selectivity on late isolates, both in a *lasR-rhlR* mutation-dependent (e.g. P01L and P08L) and -independent manner (e.g. P09L) (Fig. 3b) (Hoffman et al. 2009). The downregulation of QS may indicate selection for loss of function mutations for the entire pathway or modulation of pathway expression. To distinguish between these possibilities and to further investigate the changes in the excretion of other important chemical compounds, we performed an exo-metabolomic analysis of the secreted molecules by these lineages.

### Metabolic Distinctions during Different Evolutionary Stages

**Fig. 4.**
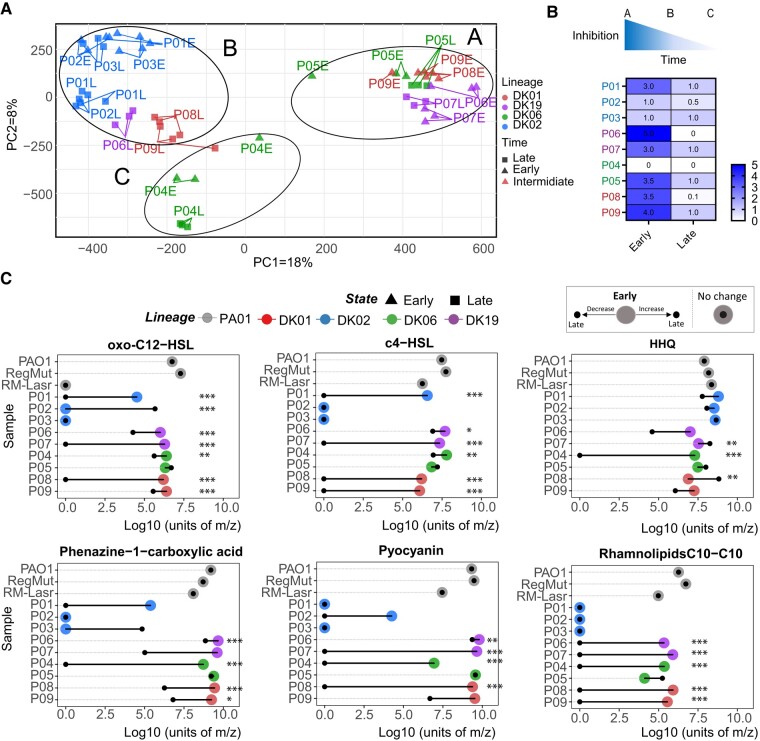
Metabolomic distribution in the different samples. a) Unsupervised PCA performed on the total exo-metabolites. Lineages are color coded (DK01 red, DK02 blue, DK06, green, and DK19 purple). b) Inhibitory effect of secreted supernatant tested on sensitive bacteria (*B. subtilis*). c) Quantification of relevant metabolite, QS regulators oxo-C12-HSL, C4-HSL, HHQ, phenazine 6-carboxylic acid, pyocyanin, and C10-C10 rhamnolipid. Secreted metabolites from early samples are represented as colored circles and the related late with a dark point. Samples coming from the same patient are connected with a line. Lineages dominating each patient are color coded (DK01 red, DK02 blue, DK06 green, and DK19 purple).

Metabolomic profiles from stationary phase cultures were analyzed by means of liquid chromatography coupled to MS (LC-MS). The molecular masses obtained were aligned and quantified for the different isolates (supplementary table S6, Supplementary Material online). Unsupervised PCA profiling of the total exo-metabolomes clearly documents different outcomes from early (cluster A) and late isolates (clusters B and C), except for DK02, which once more shows no variation between the different time points of the isolates (cluster B) (Fig. 4a). Cluster A represents bacteria, like PAO1, with normal levels of oxo-C12-homoserine-lactone (oxo-C12-HSL), 2-heptylquinolin-4(1H)-one (HHQ), phenazine, pyocyanin, and rhamnolipids (Fig. 4c). Clusters B and C show reduced or undetectable levels of oxo-C12-HSL, *N*-butanoyl-l-homoserine-lactone (C4-HSL), and HHQ, with a concomitant reduction in phenazine, pyocyanin, and rhamnolipids (Fig. 4c).

Most isolates showed only small variations in HHQ production, although statistically significant reductions were observed in 1 DK06 late isolate (Fig. 4c). Interestingly, the genes controlled by C4-HSL were downregulated in the late isolates (Fig. 3b), suggesting that the production of this QS molecule does not impact the QS network downstream, further suggesting that mutations in the central QS regulatory genes (*lasR-rhlR*) might govern (i) the transcriptional reduction of the network regulon genes and (ii) the modulation of the QS-secreted molecules.

As expected, the PAO1 *RegMutΔlasR* strain displayed a complete depletion of oxo-C12-HSL production (Fig. 4c). Moreover, it displayed a metabolome profile in between clusters A and B, with 1 to 2 Log_10_ reduction (200 times) in C4-HSL, rhamnolipids, pyocyanin, and phenazines. However, this reduction was not as drastic as in many strains from cluster B, where the levels of these molecules were nondetectable (Fig. 4c).

Since QS is associated with virulence, we tested the inhibitory properties of these secreted molecules against sensitive bacteria, and it was clear that early strains showed the highest levels of virulence (cluster A). Moreover, in all cases, the late, evolved isolates showed reduced virulence, in line with the genomic and transcriptomic information (clusters B and C) (Fig. 4b).

There was a high prevalence of some QS-regulated molecules but only small variations in the production of 4-hydroxy-3-nitroquinolin-2(1H)-one, 2-heptyl-4-hydroxyquinoline, and other quinolones from different lineages at different time points (supplementary fig. S5, Supplementary Material online). All strains showed stable excretion of the siderophore pyochelin, whereas the excretion of pyoverdine was reduced in some late isolates. Increased excretion of the amino acid norleucine was seen in the late isolates, whereas reduced excretion was observed in the early isolates compared with DK02 (supplementary fig. S5, Supplementary Material online).

In conclusion, the metabolomics data suggest that adaptation of *Pa* to human airways entails a strong selective pressure for loss of virulence-associated molecules (e.g. phenazines, pyocyanin, and rhamnolipids) and maintenance of several important ecophysiological properties (e.g. production of siderophores and quinolones). The loss of production of virulence-related molecules and modulation of the production of physiologically important molecules further suggest different functional roles for these families of molecules and, consequently, stringent modulation of their QS-mediated regulatory components.

## Discussion

We and others have previously documented that bacterial colonization and adaptation in the airways of pwCF constitute an interesting model for studying microbial evolution in complex, dynamic environments (Yang et al. 2011; Marvig et al. 2015; Dettman and Kassen 2021). With a special focus on the environmental bacterium *Pa*, the process of migration from the environment to human airways has been monitored by genotyping and phenotyping various collections of *Pa* isolates from pwCF. It has been convincingly documented that after years of bacterial colonization, *Pa* isolates derived from the patients have gone through extensive genetic and phenotypic alterations, which eventually result in the conversion of the *Pa* generalist type of organism to one that behaves much more like a niche specialist (Marvig et al. 2015).

Here, we follow 4 different lineages from pairs of isolates independently evolving in the lungs of different pwCF for periods corresponding to 35,000 to 150,000 bacterial generations. In line with previous results from our lab, we see the accumulation of mutations since the persistent infection was established (Fig. 1c; supplementary fig. S2a, Supplementary Material online). Perhaps the most important consequence of these mutations is a significant change in the genome-wide transcriptional profile for all the lineages (except for DK02). Although the strains acquire mutations at similar rates, the functional roles of these mutations may differ depending on the state of adaptation of each specific isolate and on the mutations fixed at earlier stages of the infection. Our data further suggest that when a lineage has reached an adaptation maximum, additional fixation of mutations in regulatory genes (including additional transcriptional changes) may entail fitness costs for the population, and therefore, such mutated isolates will be diluted out in the population, also illustrated by the adaptive evolution of DK02 (Yang et al. 2011). Notably, limited genomic convergence, shown as gradual accumulation of mutations during the evolution of the lineages, resulted in high convergence of the transcriptional programs. Due to the plasticity and functional redundancy of *Pa* genome, we often see that many genetic routes have similar phenotypic impact, i.e. similar global transcriptional network. Moreover, mutations that are fixed from early stages of colonization may direct the order of selection of new mutations in different arrays of genes (Marvig et al. 2015). This illustrates cases of evolutionary contingency or epistatic interactions necessary to cope with the continuously changing environment.

The transcriptional convergence program associated with the time of infection occurred in a patient-independent manner, suggesting that similar selective pressure dominates the human airway environments at late time points. Convergence of the transcription and regulatory mutational profiles was illustrated by the gain of mutations in 18 well-known pathoadaptive loci from *Pa*, mutated across the different analyzed lineages (Marvig et al. 2015) (supplementary fig. S6 and table S5, Supplementary Material online), and several late isolates presented a DK02-like transcriptome. Moreover, mutated genes could be grouped into functional categories such as envelope modifications, catabolic modulation, biofilm regulation, changes on iron response, and antibiotic-resistant genes (supplementary fig. S2c, Supplementary Material online). When a *Pa* lineage has entered the airways and developed into persistent infection, there is an array of functional mutations that need to be introduced in the genome, so the population acquires a steady state of transcriptional variation, like that seen for DK02.

One key feature that clearly distinguishes between early and late isolates is the downregulation of QS. In the investigated isolates, reduction in QS was associated with mutations in the major QS modulators *lasR-rhlR* (e.g. DK02, DK19, and DK01) genes in most of the late isolates or through mutations in the virulence modulator *gacS/retS* (e.g. *ladS* and *pprA* for DK06). In the DK19 lineage, 1 isolate (P07L) had a deletion in *lasR* with drastic effects on the transcriptional program, converting the transcriptome to that described for the DK02 lineage (Fig. 1d; supplementary fig. S7, Supplementary Material online). But, the *lasR-rhlR* mutation alone could not explain the drastic reduction of HSL and virulence-related moieties observed in late isolates (e.g. phenazines), as shown for the PAO1 RegMutΔlasR strain with a *lasR* deletion, which shows a reduction of QS, less drastic than what is observed in the late isolates. It is therefore likely that the mutational profiles acquired during persistent infection may comprise loss of function mutations within the regulatory networks implicated in the modulation of QS.

QS downregulation was observed mainly in isolates, which were obtained many years after infection of the patient’s airways (e.g. 1 to 3 decades). This may suggest that at early times, QS may be important for the establishment of the infection. In fact, reduction of QS is usually associated with increased probability of persistent infection (Hoffman et al. 2009). During the progress of the infection in time, the CF airways show biotic and abiotic physiological variations. Usually, there is a dysregulated immune system (e.g. increase the population of neutrophils and immune cells) and changes in the biotic environment in the lung, associated with decreases in microbiological diversity and dominance of 1 or a few opportunistic bacteria (Rossi et al. 2021). Moreover, expression of virulence factors and QS molecules may be energetically costly, and due to fitness pressure, loss-of-function mutations are acquired when the population diversity declines. Therefore, it is possible that the evolutionary convergence observed in the isolates could be related to a similar selective force governing the CF lung environment at a late time as a response to an ecophysiological variation (Luján et al. 2022).

Although the production of acyl-homoserine lactone and the transcriptional network modulated by AHLs was eliminated in most of the late isolates, some QS-regulated molecules were synthesized, probably independently of the QS network. Among the molecules produced by the *Pa* isolates at late time points, siderophores and quinolones could be associated with specific functional roles of these molecules. For example, siderophores, commonly known as metal chelators implicated in iron and other metals’ homeostasis, were produced by all the isolates, at least 1 type (e.g. pyoverdine vs. pyochelin). Moreover, HQNO and other AQNO quinolones are redox molecules implicated in the modulation of the immune response and virulence factors for other bacterial warfare (Lin et al. 2018). It is possible that the selective value of AQNOs is related to immune modulation more than to virulence, as the more virulent-associated molecules phenazines/pyocyanin and rhamnolipids (Glasser et al. 2014) showed a drastic decrease with time. In summary, both iron homeostasis modulation and probably immune modulation may be key features for the adaptation of *Pa*.

Finally, associating evolutionary data with patient information could be used as a proxy for the development of biomarkers to determine the patient's prognosis and/or disease development. We believe that evolutionary studies like the one presented here could help pinpoint genomic determinants associated with pathway-specific selectivity, providing an easier genomic–phenotypic association. Moreover, it could provide a proper biomarker of the infection stages and improve treatment options for the patients.

## Materials and Methods

### *Pa* CF Isolate Collection, Ethics Approval, and Consent to Participate

Clinical isolates were obtained from sputum samples from 9 pwCF attending or that have attended the Copenhagen Cystic Fibrosis Center at University Hospital Rigshospitalet, Copenhagen, Denmark. Sputum sampling is part of routine clinical visits in the CF clinic and is not performed for the purposes or intent of this research. The use of the isolates was approved by the local ethics committee of the Capital Region of Denmark (Region Hovedstaden; registration number H-21078844). Isolation and identification of *Pa* from sputum were carried out as previously described (Holm et al. 2021). The *Pa* collection included a pair of isolates from each of the patients, 1 taken at the beginning of the chronic infection and 1 taken after a period of 15 to 40 yr depending on the patient/lineage. The time in which a pair of isolates was collected is summarized in Table 1.

### Laboratory Bacterial Strains

*Pa* reference strain PAO1 was used in this study together with 2 isogenic mutants (Regmut and RegmutΔlasR) previously constructed in the lab associated to DK02 evolutionary history (Damkiær et al. 2013). Regmut, consisted of a triple mutant based on specific *mucA-*, *algT-*, and *rpoN* alterations, and RegmutΔlasR included an extra deletion of *lasR* gene, giving a quadruple mutant configuration.

### Comparative Genomics

Genomic DNA was extracted and purified from overnight (ON) liquid cultures of bacterial single colonies using a DNeasy Blood and Tissue kit (Qiagen). Genomic DNA libraries were prepared using a Nextera XT DNA Library Prep Kit (Illumina), and libraries were sequenced on either a MiSeq (69 libraries) or NextSeq 500 platforms (84 libraries), generating 250- or 150-bp paired-end sequencing reads, respectively. Sequencing reads were trimmed, and low-quality reads and potential contamination from adapters were removed using Trimmomatic (v0.35) tool (Bolger et al. 2014). Genomic analysis was conducted by BacDist (Gabrielaite and Maanmi 2020) to identify genomic variants relative to PAO1 reference genome (NCBI: NC_002516.2). BacDist filtered mutations to only retain variants with a mapping quality of at least 50, a minimum coverage of 10 and a minimum fraction of 50% of reads supporting the variant, excluding the mutations shared by all isolates (>80% of reads supporting the variant) at a given position. Variations unique to each clone belonging to the same lineage were used to determine potential transmissions and to estimate an average evolutionary distance.

### Phylogeny Reconstruction of CF Isolates

Evolutionary analyses were conducted in MEGA11 (Tamura et al. 2021). For this purpose, concatenated sequences of only the SNPs of the 19 CF isolates were aligned to the positions of the nucleotides in the genome of the reference strain PAO1. There were a total of 62,987 positions in the final dataset. The evolutionary history was inferred by using the maximum likelihood method and general time reversible model (Tamura et al. 2021) and 500 bootstraps were set for analysis confidence. The bootstrap tree is shown.

### Estimation of Bacterial Evolution Rates

Evolution rates were assessed as the number of SNPs per genome size per generation as previously (Yang et al. 2011). Generation times, assembly size, and estimation of bacterial evolution are summarized in supplementary table S2, Supplementary Material online. For SNPs, the number of missense, stop, and synonymous mutations accumulated between the pairs of early and late isolates was used. For the estimation of bacterial generations, we calculated the growth rate of each isolate in SCFM media that gave us an average doubling time of 140.04 ± 49 min. This value was already on the range of previously published data from in vivo doubling time ratios (Yang et al. 2008). The number of bacterial generations elapsed over time was calculated as the sum of generations from the year of isolation of the early to the late isolate. As a proxy of genome size for each lineage, we assembled the genomes of 1 single pair of isolates: for DK01(P09I-P09L), DK02 (P02E-P02L), DK06 (P04E-P04L), and DK19 (P07E-P07L). For this, paired-end reads were assembled into contigs using Spades (Bankevich et al. 2012) and quality was evaluated with QUAST (Gurevich et al. 2013).

### Library Preparation and RNA Sequencing

Single colony cultures were grown in SCFM media (inoculation with OD_600_ = 0.05) at 37 °C under shaking conditions (200 rpm) to mid-exponential phase (OD_600_ = 0.35 to 0.5). RNA was extracted with RNeasy Mini Kit (Qiagen) according to the manufacturer's instructions. Transcription was blocked by applying RNA Protect Bacteria solution (Qiagen). RNA was quality checked using an Agilent Bioanalyzer 2100 (Agilent Technologies) (RNA integrity number > 9). For all other samples used in this study, 100 ng of total RNA was used as input for the generation of RNA libraries with Illumina Stranded Total RNA Prep, Ligation with Ribo-Zero Plus Kit, and following the manufacturer’s instructions. After quality and size distribution check on DNA HS chips on an Agilent Bioanalyzer 2100 machine, libraries were pooled in equimolar amounts and sequenced on an Illumina NextSeq 500 machine. An average of 10 to 15 million reads with 2 × 75-bp-long reads were obtained per sample. Mapping was performed using the PA14 genome as a reference.

### Comparative Transcriptomics

Reads were trimmed, and low-quality reads and potential contamination from adapters were removed using Trimmomatic (v0.35) tool (Bolger et al. 2014). Reads were further processed using SortMeRNA tool (v2.1) (Kopylova et al. 2012) to remove reads generated from residual rRNA transcripts. As DK19 is the PA14 lineage, and to have a better read alignment, reads were mapped against *UCBPP-PA14* genome (NCBI: NC_008463.1) using BWA-MEM algorithm, and duplicated reads were marked using Picard tools. Reads mapping on each annotated coding sequence were counted using htseq-count v0.7.2 (Anders et al. 2013) imported and processed in RStudio (RStudio Team 2020).

### Transcriptional Analysis

Counts were normalized using log_2_-negative binomial transformation performed using the rld transformation function contained in the R package DESeq2 (Love et al. 2014) with the option blind set as “true.” Normalized counts were used to evaluate whole transcriptome similarities using hierarchical clustering analysis (HCA), PCA, and k-mean clustering on selected normalized data. HCA was performed using the function “pheatmap” in the R package complexheatmap (Gu et al. 2016). Pearson's correlation coefficient (cor()) was applied on the normalized data as a distance method. PCA on normalized reads counts was performed using prcomp() function with the scale option set as “false.” DEG analysis between transcriptomes was performed using the R package DESeq2, considering statistically significant genes with a Log_2_(fold change) ≥ |2| and an adjusted *P* ≤ 0.01 (Love et al. 2014). DEGs were inspected and functional class enrichment was performed using the provided the R package ClusterProfiler (Yu et al. 2012) for KEGG and GO categories with default settings. The convergent enriched pathways, similar up/downregulation for each pair of samples, were evaluated by quantifying the frequency that enriched categories appear in at least 2 pairs of samples.

### Comparative Metabolomics

Single colonies were isolated on Luria Bertani (LB) agar plates and transferred to a tube containing 2 mL SCFM + GlcNAg medium. This preculture was normalized to OD_600_ = 0.05 using fresh medium and added to a flask. Samples were incubated at optimal temperature/shaking conditions. Bacterial cultures were isolated to the stationary phase when no OD variations were observed, OD_600_ = 2 to 3, *t* = 24 h for early strains and 36 h for late isolates. For all the isolates, 2 mL of actively growing bacteria were centrifuged (6,000 × *g*, 10 min), and the supernatant and pellet were used for further experimentation. The supernatant was 0.22 µm filtered and concentrated on a speed vacuum at room temperature. Dried pellets were stored at −80 °C. The pellets were concentrated 10 times and resuspended 50:50 MQ water:methanol. The samples were run on a Vanquish Duo UHPLC binary system (Thermo Fisher Scientific) coupled to IDX-Orbitrap Mass Spectrometer (Thermo Fisher Scientific, USA). The compound separation was achieved in reverse phased using a Waters ACQUITY BEH C18 (10 cm × 2.1 mm, 1.7 μm) column equipped with an ACQUITY BEH C18 guard column kept at 40 °C and mobile phase consisting of MilliQ water + 0.1% formic acid (A) and acetonitrile + 0.1% formic acid (B) at a flow rate of 0.35 mL/min as previously described (Kildegaard et al. 2021). The MS acquisition was set in positive-heated electrospray ionization mode with a voltage of 3,500 V acquiring in full MS/MS spectra (data-dependent acquisition-driven MS/MS) in the mass range of 70 to 1,000 Da. The DDA acquisition settings were as follows: automatic gain control target value was set at 4 × 10^5^ for the full MS and 5 × 10^4^ for the MS/MS spectral acquisition; the mass resolution was set to 120,000 for full scan MS and 60,000 for MS/MS events. Precursor ions were fragmented by stepped high-energy collision dissociation using collision energies of 20, 40, and 60. All the analyses were carried out in biological triplicates. LC-MS chromatograms were aligned and quantified using Mzmine with default parameters (Pluskal et al. 2010). Masses were further processed in R. Missing values were given a 0 value. For unsupervised clustering, acquired masses were normalized in R with a negative binomial normalization, applying a variant stabilizing normalization inside the DESeq2 package (Love et al. 2014), with the option blind set as “true.” Molecular masses were confirmed by comparing retention time to commercial standards (e.g. oxo-C12-HSL, phenazines, pyocyanin, and rhamnolipids), development of pathways synthetic mutants (e.g. deletion *pqsABC* and *pvdI* quinolones and pyoverdine, respectively), and virtual libraries (e.g. rhamnolipids) (Wang et al. 2016). Masses were validated by analyzing their fragmentation profile to that previously stored in the GNPS Library (Wang et al. 2016). Statistical analyses were performed with unpaired Log10 multi-*t*-test inside GraphPad. Differences were considered statistically significant at *P* < 0. 05.

### Inhibitory Assay

A preculture of *Bacillus subtilis* was grown ON and normalized to OD_600_ = 0.01. Two hundred microliters of cultures were spread to LB agar. The plates were dried and Whatman paper discs were attached. Then, 5 µL of the concentrated bacteria filtered and concentrated 10 µL supernatant was added to the discs. Plates were incubated at 37 °C ON. As a positive control, 1,500 µg of ciprofloxacin was added (5 µL at 300 µg/mL) and, as a negative control, fresh concentrated SCFM medium.

### Antibiotic Susceptibility Testing

Microdilution test was carried out as described by the EUCAST guidelines (The European Committee on Antimicrobial Susceptibility Testing 2023). Briefly, bacterial isolates were grown ON at 37 °C in Müller–Hinton broth. The following day, inoculum was corrected to seed 5 × 10^5^ CFU/mL in microtiter plates containing serial fold dilutions of either ceftazidime, tobramycin, or ciprofloxacin using Müller–Hinton broth as diluting media. MIC was calculated as the lowest concentration of antimicrobial agent that completely inhibited the growth of the organism as detected by the unaided eye. The experiment was performed in duplicates for each isolate.

### Frequency of Mutation to Rifampicin Resistance

Bacterial isolates were grown ON in 3 mL LB medium, and then 1 mL was centrifuged at 3,000 rpm for 10 min and resuspended in 100 μL LB medium. Serial fold dilutions were plated on LB plates containing 300 μg/mL rifampin and on LB plates without rifampicin. Numbers of CFU were counted after incubation at 37 °C for 48 h (24 to 36 h for fast growers and 48 h for slow growers). Mutation frequency was calculated based on the number of colonies resistant to rifampicin in every 10^8^ viable cells (counted on LB plates). An isolate was considered a hypermutator if the mutation frequency after exposure to rifampin was 20 times higher than the mutation frequency of the reference strain PAO1 (Oliver et al. 2000). Frequencies were determined from 2 independent experiments.

## Supplementary Material

msae022_Supplementary_Data

## Data Availability

Whole-genome sequences of isolates P01E, P01L, and P03E were downloaded from previously published data from the Sequence Read Archive (SRA) study ERP002277. The same applies for isolates P05E and P06E, which were obtained from the SRA study with accession number ERP004853. Raw sequences of the rest of the WGS and RNA-seq have been deposited in the SRA under BioProject ID PRJNA991306. See supplementary table S7, Supplementary Material online for the accession codes of individual isolates data.
