# Supplementary material for: Adaptive Evolution of Pseudomonas aeruginosa in Human Airways Shows Phenotypic Convergence Despite Diverse Patterns of Genomic Changes
Source: Mol Biol Evol. 2024 Feb 14;41(2):msae022. doi: 10.1093/molbev/msae022 (PMC10883414; doi:10.1093/molbev/msae022)
Supplement: msae022_Supplementary_Data [file msae022_supplementary_data.zip › FigureS2-remap.pdf]

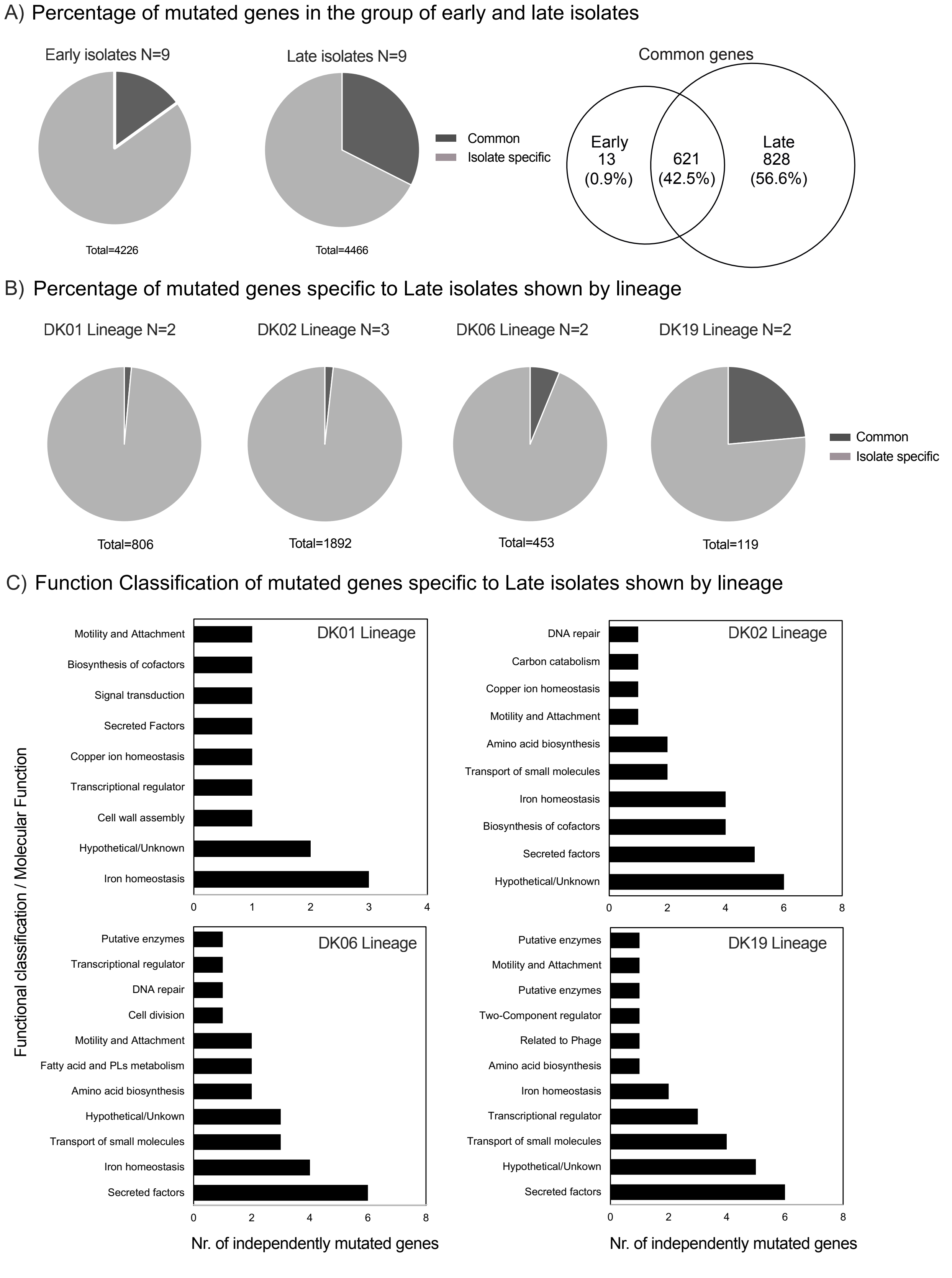
A) Percentage of mutated genes in the group of early and late isolates
Early isolates N=9
Total=4226
Late isolates N=9
Total=4466
Common
Isolate specific
Common genes
Early
13
(0.9%)
621
(42.5%)
Late
828
(56.6%)
B) Percentage of mutated genes specific to Late isolates shown by lineage
DK01 Lineage N=2
Total=806
DK02 Lineage N=3
Total=1892
DK06 Lineage N=2
Total=453
DK19 Lineage N=2
Total=119
Common
Isolate specific
C) Function Classification of mutated genes specific to Late isolates shown by lineage
DK01 Lineage
Motility and Attachment
Biosynthesis of cofactors
Signal transduction
Secreted Factors
Copper ion homeostasis
Transcriptional regulator
Cell wall assembly
Hypothetical/Unknown
Iron homeostasis
0
1
2
3
4
DK02 Lineage
DNA repair
Carbon catabolism
Copper ion homeostasis
Motility and Attachment
Amino acid biosynthesis
Transport of small molecules
Iron homeostasis
Biosynthesis of cofactors
Secreted factors
Hypothetical/Unknown
0
2
4
6
8
DK06 Lineage
Putative enzymes
Transcriptional regulator
DNA repair
Cell division
Motility and Attachment
Fatty acid and PLs metabolism
Amino acid biosynthesis
Hypothetical/Unkown
Transport of small molecules
Iron homeostasis
Secreted factors
0
2
4
6
8
DK19 Lineage
Putative enzymes
Motility and Attachment
Putative enzymes
Two-Component regulator
Related to Phage
Amino acid biosynthesis
Iron homeostasis
Transcriptional regulator
Transport of small molecules
Hypothetical/Unkown
Secreted factors
0
2
4
6
8
Functional classification / Molecular Function
Nr. of independently mutated genes
Nr. of independently mutated genes
