# Supplementary figures and images for: Adaptive Evolution of Pseudomonas aeruginosa in Human Airways Shows Phenotypic Convergence Despite Diverse Patterns of Genomic Changes
Source: Mol Biol Evol. 2024 Feb 14;41(2):msae022. doi: 10.1093/molbev/msae022 (PMC10883414; doi:10.1093/molbev/msae022)

# Percentage of pwCF infected with each lineage

Top 10 most-abundant clone-types (55.6% of total patients)

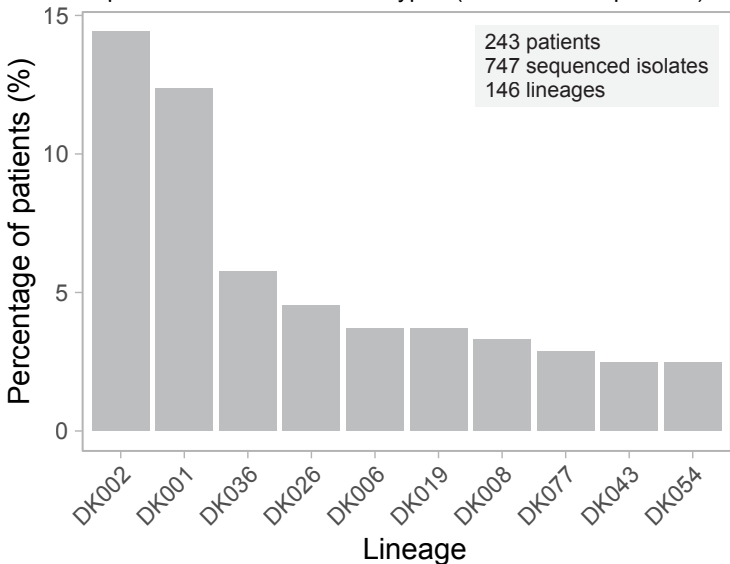

Supplement: msae022_Supplementary_Data [file msae022_supplementary_data.zip › FigureS1.pdf]

A) **Transcriptional variability**

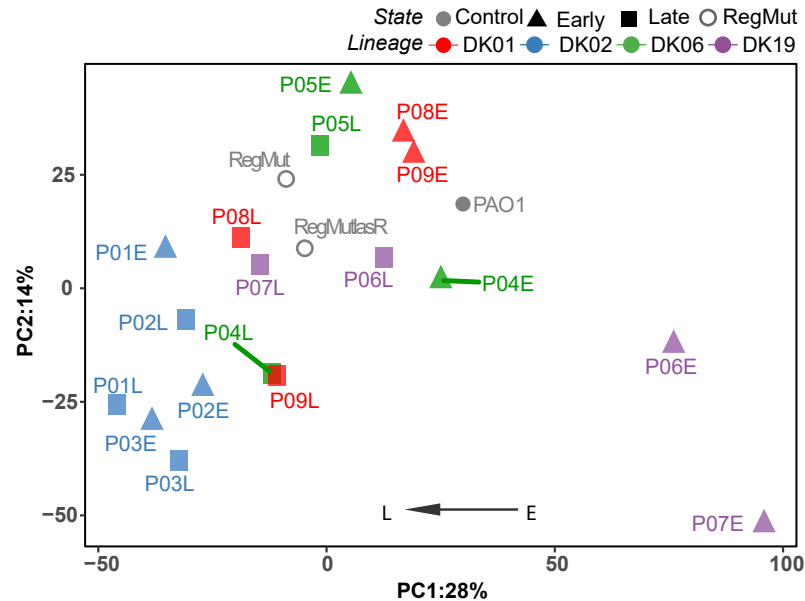

B) **Transcriptional similarity**

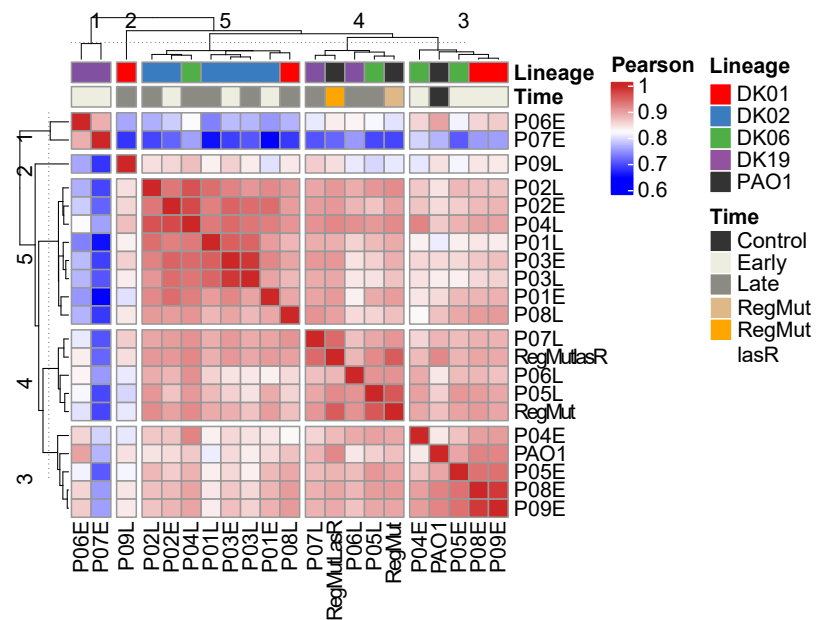

Supplement: msae022_Supplementary_Data [file msae022_supplementary_data.zip › FigureS3.pdf]

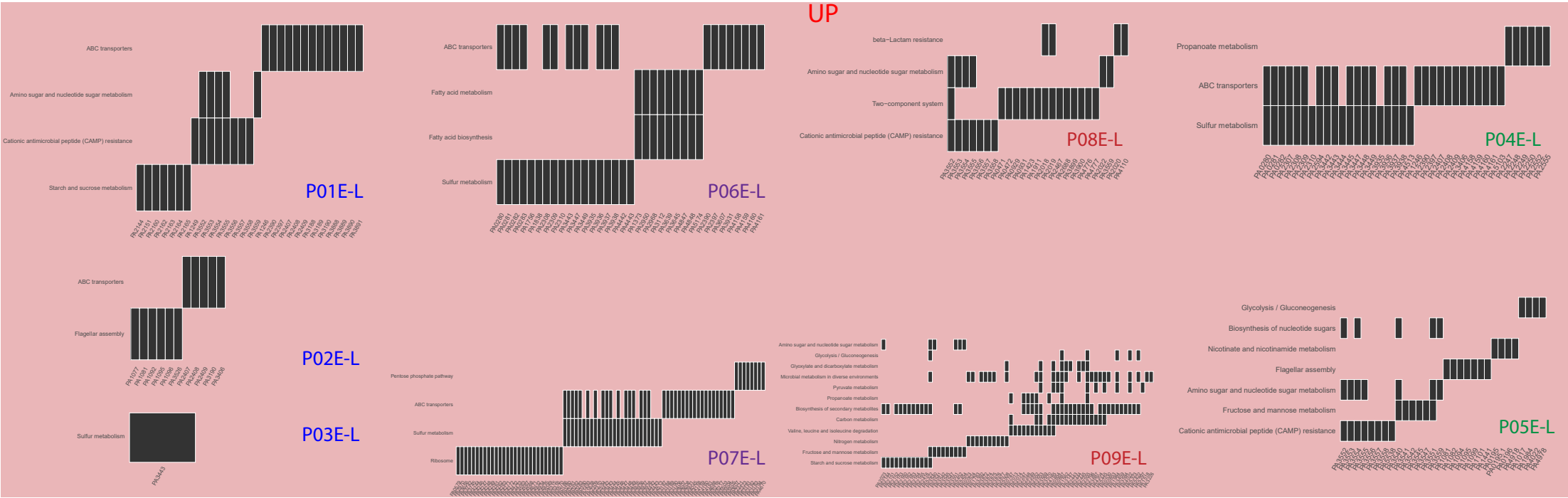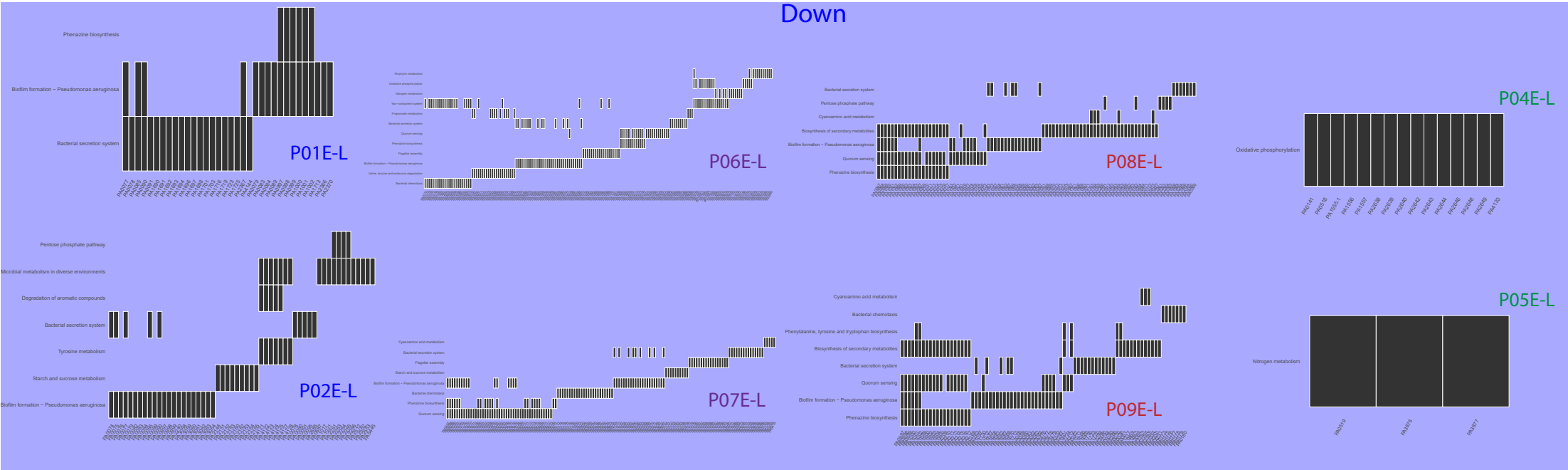

Supplement: msae022_Supplementary_Data [file msae022_supplementary_data.zip › FigureS4.pdf]

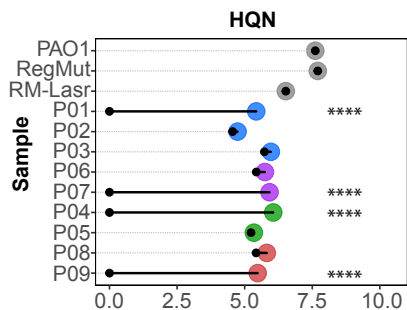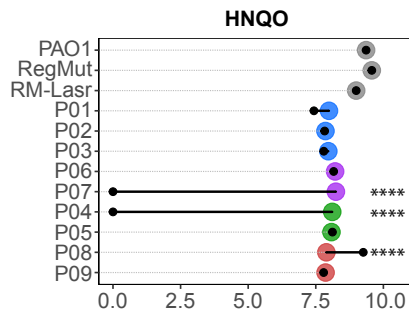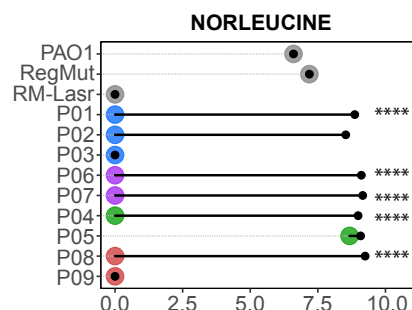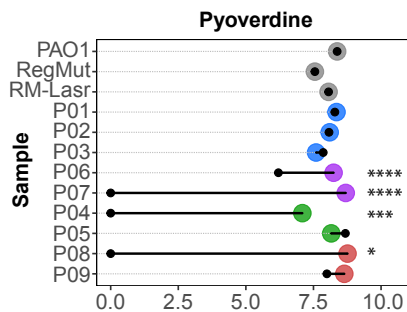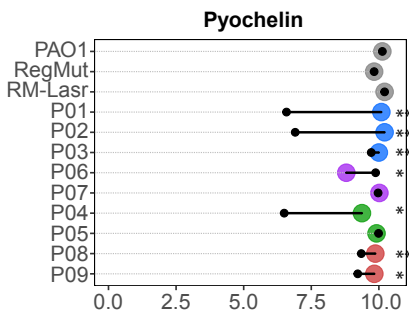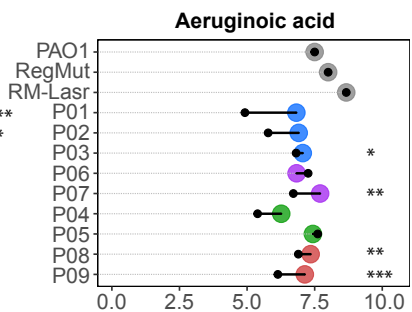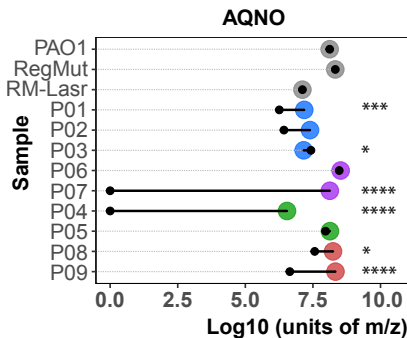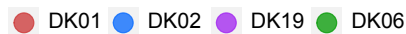

Supplement: msae022_Supplementary_Data [file msae022_supplementary_data.zip › FigureS5.pdf]

# Mutations in Pathoadaptive genes in the CF collection

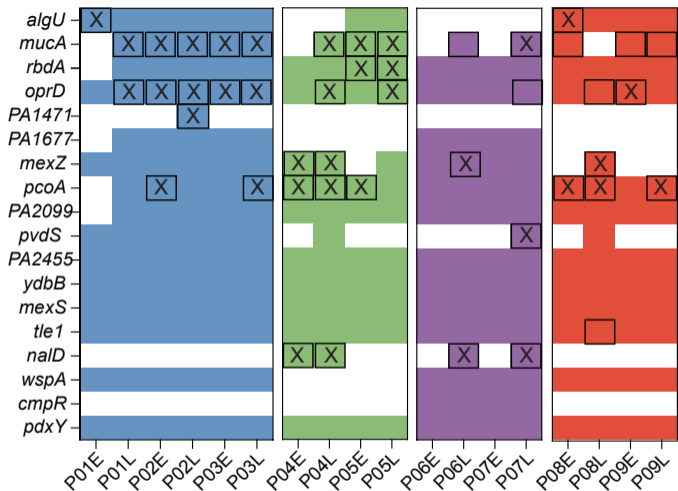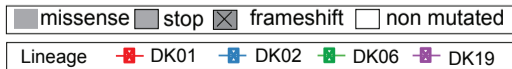

Supplement: msae022_Supplementary_Data [file msae022_supplementary_data.zip › FigureS6-remap.pdf]
