# Supplementary material for: Adaptive Evolution of Pseudomonas aeruginosa in Human Airways Shows Phenotypic Convergence Despite Diverse Patterns of Genomic Changes
Source: Mol Biol Evol. 2024 Feb 14;41(2):msae022. doi: 10.1093/molbev/msae022 (PMC10883414; doi:10.1093/molbev/msae022)
Supplement: msae022_Supplementary_Data [file FigureS7.pdf › FigureS7.pdf]

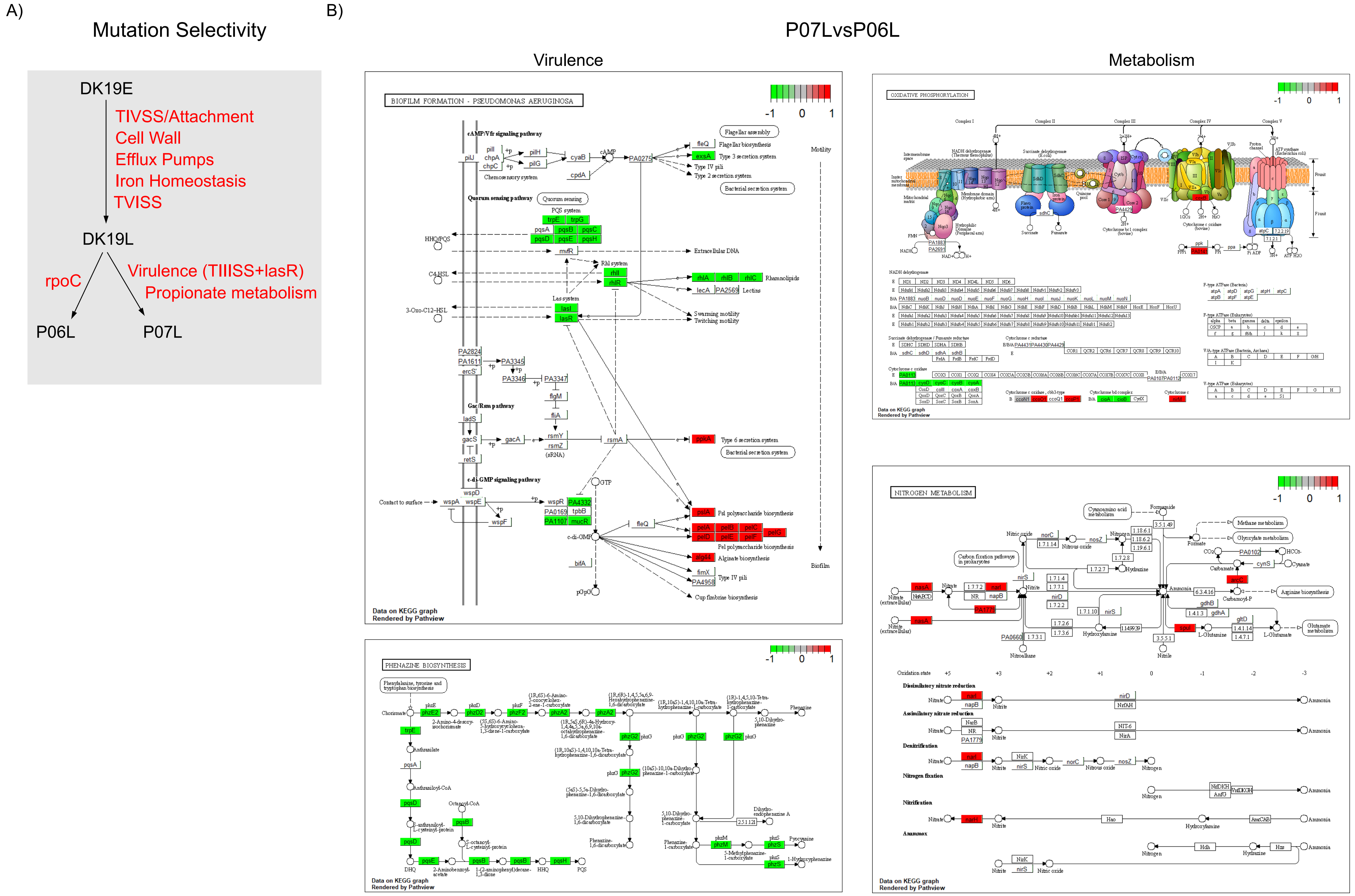
A)
Mutation Selectivity
DK19E
TIVSS/Attachment
Cell Wall
Efflux Pumps
Iron Homeostasis
TVISS
DK19L
rpoC
Virulence (TIIISS+lasR)
Propionate metabolism
P06L
P07L
B)
P07LvsP06L
Virulence
Metabolism
BIOFILM FORMATION - PSEUDOMONAS AERUGINOSA
-1 0 1
Data on KEGG graph
Rendered by Pathview
PHENAZINE BIOSYNTHESIS
-1 0 1
Data on KEGG graph
Rendered by Pathview
OXIDATIVE PHOSPHORYLATION
-1 0 1
Data on KEGG graph
Rendered by Pathview
NITROGEN METABOLISM
-1 0 1
Data on KEGG graph
Rendered by Pathview
